# Characterization of the small RNA component of leaves and fruits from four different cucurbit species

**DOI:** 10.1186/1471-2164-13-329

**Published:** 2012-07-23

**Authors:** Guru Jagadeeswaran, Padma Nimmakayala, Yun Zheng, Kanchana Gowdu, Umesh K Reddy, Ramanjulu Sunkar

**Affiliations:** 1 Department of Biochemistry and Molecular Biology, Oklahoma State University, Stillwater, OK, 74078, USA; 2 Department of Biology and Gus R. Douglass Institute, West Virginia State University, Institute, WV, 25112, USA; 3 Institute of Developmental Biology and Molecular Medicine School of Life Sciences, Fudan University, Shanghai, 200433, China

## Abstract

**Background:**

MicroRNAs (miRNAs) are a class of non-coding small RNAs involved in post-transcriptional regulation of gene expression critical for plant growth and development, stress responses and other diverse biological processes in plants. The *Cucurbitaceae* or cucurbit family represents some of economically important species, particularly those with edible and medicinal fruits. Genomic tools for the molecular analysis of members of this family are just emerging. Partial draft genome sequence became available recently for cucumber and watermelon facilitating investigation of the small RNA component of the transcriptomes in cucurbits.

**Results:**

We generated four small RNA libraries from bottle gourd (*Lagenaria siceraria*), *Cucurbita moschata, Cucurbita pepo*, and, watermelon (*Citrullus lanatus var. lanatus*) in order to identify conserved and novel lineage specific miRNAs in these cucurbits. Deep sequencing of small RNA libraries from these species resulted in 1,597,263, 532,948, 601,388, and 493,384 unique sRNA reads from bottle gourd, moschata, pepo and watermelon, respectively. Sequence analysis of these four libraries resulted in identification of 21 miRNA families that are highly conserved and 8 miRNA families that are moderately conserved in diverse dicots. We also identified 4 putative novel miRNAs in these plant species. Furthermore, the tasiRNAs were identified and their biogenesis was determined in these cucurbits. Small RNA blot analysis or q-PCR analyses of leaf and fruit tissues of these cucurbits showed differential expression of several conserved miRNAs. Interestingly, the abundance of several miRNAs in leaves and fruits of closely related *C. moschata* and *C. pepo* was also distinctly different. Target genes for the most conserved miRNAs are also predicted.

**Conclusion:**

High-throughput sequencing of small RNA libraries from four cucurbit species has provided a glimpse of small RNA component in their transcriptomes. The analysis also showed considerable variation within four cucurbit species with regards to expression of individual miRNAs.

## Background

Although transcriptional gene regulation is the most important mode of gene regulation, miRNA-dependent post-transcriptional gene regulation is also absolutely necessary for completion of life cycle in plants as the disruption of either the miRNA biogenesis or function is lethal. Thus, identification of miRNAs and their targets from various plant species of agricultural importance is not only vital as part of basic biology but might also have biotechnological applications. MicroRNA genes are transcribed by RNA Polymerase II and the resulting pre-miRNA transcript can adopt a hairpin-like structure. These structures serve as substrates for Dicer-like 1 (DCL1), an endoribonuclease that precisely severs the hairpin-like structure and releases 21-nt long miRNA:miRNA* duplex with 2-nt overhangs at the 3′ends. Although DCL1 is a major determinant for processing of miRNAs, several other proteins such as Hyponastic Leaves1 (HYL1), Serrate (SE), Dawdle (DDL) and Cap-binding protein (CBP) are also important for processing of miRNAs from its hairpin-like precursor ([1-6]. The 21 nt miRNA:miRNA* duplex is methylated at the 3′ends by the Hua Enhancer 1 (HEN1). The duplex is then exported to the cytosol, where it can be loaded into RNA-Induced Silencing Complex (RISC). The miRNA in the RISC serves as guide molecule to identify its target mRNA, which will be either degraded or prevented from being translated [7-10].

Approximately 21 miRNA families are known as highly conserved among the angiosperms (dicots and monocots) and 8 and 6 of these miRNA families conserved even in gymnosperms and bryophytes, respectively [11,12]. Because of the sequence conservation, identification of conserved miRNAs is not a difficult task provided sufficient genomic/EST resources are available for a plant species in question [13,14]. Even in the absence of genomic/EST resources, conserved miRNAs can be identified by expression analysis using probes designed to detect conserved miRNAs. Besides conserved miRNAs, a large number of miRNA families have been identified in diverse plant species, some of which are conserved in closely related species or species-specific, which are referred to as ‘young miRNAs’ [12]. Only a minor portion of these young miRNAs appears to be functional, whereas the majority of them appear to be non-functional and eventually dissipate, and only few of them will be integrating into gene regulatory networks [12,15]. Although functionally such non-conserved miRNAs seem less significant, their identification is important to trace the birth and death of miRNAs in a specific plant lineage [12].

MicroRNAs have been reported from several model or crop plant species [16-23], but little is known about the miRNA component in the family of *Cucurbitaceae*. *Cucurbitaceae* is known to have 90 genera and 700 species, out of which there are several domesticated species for food that includes *Citrullus lanatus* (watermelon), *Cucumis sativus* (cucumber), *Cucumis melo* (melon), *Lagenaria siceraria* (bottle gourd) and *Cucurbita* (squash & pumpkin) [24-27]. Little is known about the small RNA component of the transcriptome in a Cucurbitaceae member. Only recently, miRNAs in cucumber (*Cucumis sativus*) and melon (*Cucumis melo*) have been analyzed using relatively small number of reads [28,29]. Here we report high-throughput sequencing of small RNA libraries from the four different cucurbit species. The analysis identified 21 highly conserved miRNA families, and 8 miRNA families that are only moderately conserved in various dicots. Analysis of small RNA libraries from four cucurbit species facilitated identification of 4 putative novel miRNAs based on their recovery in at least two different cucurbit genera. Cucurbit small RNAs with similarity to Arabidopsis TAS3 siRNAs were identified and their biogenesis was also determined. Furthermore, we predicted target genes for the most conserved miRNAs with the available watermelon, pepo and melon genomes.

## Results and discussion

### Sequence analysis and annotation

We generated four small RNA libraries from the pooled RNA isolated from different tissues (equimolar concentration from leaf, stem, as well as flesh, rind and placenta of the fruits) from bottle gourd (*Lagenaria siceraria* [accession Grif 1617 collection from India])*, Cucurbita moschata* (accession Grif 14244 Early Butternut) (referred to as moschata hereafter) *Cucurbita pepo* (accession NSL98075 Table King) (referred to as pepo hereafter), and watermelon (*Citrullus lanatus var. lanatus*) (PI 438676 Charleston Grey). Plants were grown under greenhouse conditions (16-h light). Leaf and stem tissues were collected from three-week-old seedlings. Fruit tissues were collected 10 days after pollination. We pooled the RNA from different vegetative and fruit tissues in order to uncover maximum number of conserved and novel miRNAs that are expressed in diverse tissues or different cell-types. The libraries were sequenced using Illumina-GAII analyzer. Initially the 5′ adapter from the raw reads was trimmed and small RNAs ranging between 18–30 nucleotides extracted. We have obtained 29,640,508, 10,491,849, 13,063,242, and 7,079,207 total reads represented by 1,597,263, 532,948, 601,388, and 493,384 unique small RNA reads from bottle gourd, moschata, pepo and watermelon, respectively (NCBI GEO_GSE38176) (Table 1). The unique small RNAs have been used to identify conserved miRNAs by mapping the sequences with the miRNA homologs available at the miRBase (version 16, obtained from http://microrna.sanger.ac.uk/sequences/ftp.shtml). The remaining unique sequences were used to identify the degradation products from rRNA, tRNA, snRNA and snoRNAs, by searching against the non-coding RNA database. The sequences that did not map to noncoding RNAs were used to identify novel miRNAs pertaining to four cucurbit species.

**Table 1:** Small RNA libraries in different cucurbit species

	***Bottle gourd***		***Moschata***		***Pepo***		***Watermelon***	
	**Unique**	**Total**	**Unique**	**Total**	**Unique**	**Total**	**Unique**	**Total**
ncRNAs	249,263	12,135,459	103,168	4527,726	113,948	5,678,005	43,524	2,418,965
miRBase	27,287	726,073	11,272	223,633	12,952	270,779	5,448	348,849
mRNAs	80,675	338,251	26,760	99,859	30,304	118,907	26,015	134,651
Repeats	222,416	2,156,450	75,075	581,693	84,641	696,941	49,065	440,122
Genome	1,017,622	14,284,275	316,673	5,058,938	359,543	6,298,610	369,332	3,736,620
Total	1,597,263	29,640,508	532,948	10,491,849	601,388	13,063,242	493,384	7,079,207

**Figure 1:**
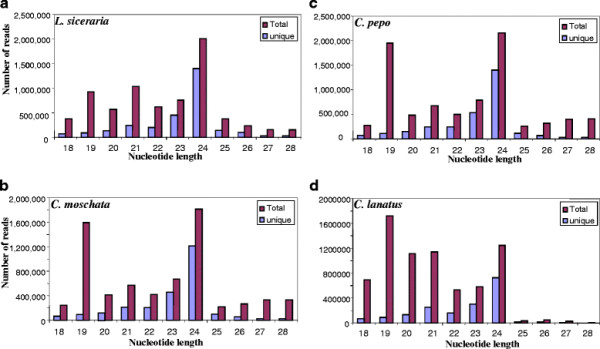
Abundance of different sizes of small RNAs (length 18–28 nt) in total and unique reads from small RNA libraries a) bottle gourd b) moschata c) pepo d) watermelon.

Plant small RNA population is predominantly represented by two different sizes, i.e., 21-nt and 24-nt, which are typical of DCL1/DCL2/DCL4 and DCL3 processed small RNAs, respectively [15]. In general, the peak represented by 24-nt size class is greater than the 21-nt size class in several plant species [15,22,30,31] with a few exceptions, as in grapevine, where the 21-nt size class is the major peak and 24-nt class is the minor peak [32]. The abundance of total and unique small RNAs of 18 to 28 nucleotides in different cucurbits is shown in Figure 1. Except watermelon, small RNA libraries of the other three cucurbits had the highest peak representing 24-nt size class. The second highest peak representing 21-nt size class was found only in the bottle gourd small RNA library, whereas 19-nt size class represented the second highest peak in three other cucurbits (Figure 1). In fact, 19-nt size class was the second highest peak in pepo and moschata whereas this size class was even greater than 24-nt size class in watermelon. The abundance of unique reads belonging to 19-nt class is much smaller in all the libraries indicating their greater redundancy. Closer inspection of 19-nt sequences suggested that some of the highly abundant sequences arise from tRNA-derived fragments from the 5′end. In Arabidopsis, it has been shown that 19-nucleotide small RNAs are also processed from 5′ ends of Gly-tRNA and Asp-tRNA [33]. Interestingly these t-RNA-derived small RNAs differentially accumulated; high levels in roots but very low levels in shoots. Similarly, in HeLa (mammalian) cell lines, t-RNA-derived small RNAs have been shown to be dependent on Dicer, both *in vitro* and *in vivo*[34]. It has also been demonstrated that t-RNA-derived small RNAs are not random byproducts of degradation or biogenesis of tRNAs [35]). Taken together, 19 nt small RNAs derived from t-RNAs in cucumber may not represent simple degradation products, but may have biological functions.

With regards to unique reads, the distribution of size classes was uniform across the species, where 24-nt size class and 23-nt size class ranked first and second respectively in all 4 libraries (Figure 1). Small RNAs of 20-21-nt size class represents largely miRNAs. Recently, Martinez et al. reported small RNAs from cucumber (*Cucumis sativus* belonging to Cucurbitaceae family) by analyzing 38,787 unique small RNAs [29], which is far less compared to the current study in which several millions of small RNAs from 4 different cucurbits were analyzed. Size distribution of small RNAs in their library was typical of a plant small RNA library with two peaks one each at 21-nt and at 24-nt. Martinez et al. constructed small RNA libraries from the leaves and phloem exudates [29], whereas we used pooled RNA from leaves, stems and three different fruit tissues. Mix of diverse tissues used in this study, particularly the inclusion of fruit tissues might have contributed to the variation in size distribution including the observed peak at 19-nt size class and deserves further study.

### Identification of conserved miRNAs in different cucurbit species

Based on their functional analysis, conserved miRNAs appear to be involved in almost all aspects of plant growth and development [8], as well as biotic and abiotic stress responses [36-38]. Our analysis of small RNAs has identified all 21 conserved miRNA families in four different cucurbits as expected (Additional file 1). Additionally, miR894 and miR2111 were recovered in all 4 cucurbits, whereas miR158, miR824, miR827, miR858, miR2916 and miR2950 that are moderately conserved in some of the dicots could be identified only in watermelon (Additional file 1).

**Figure 2:**
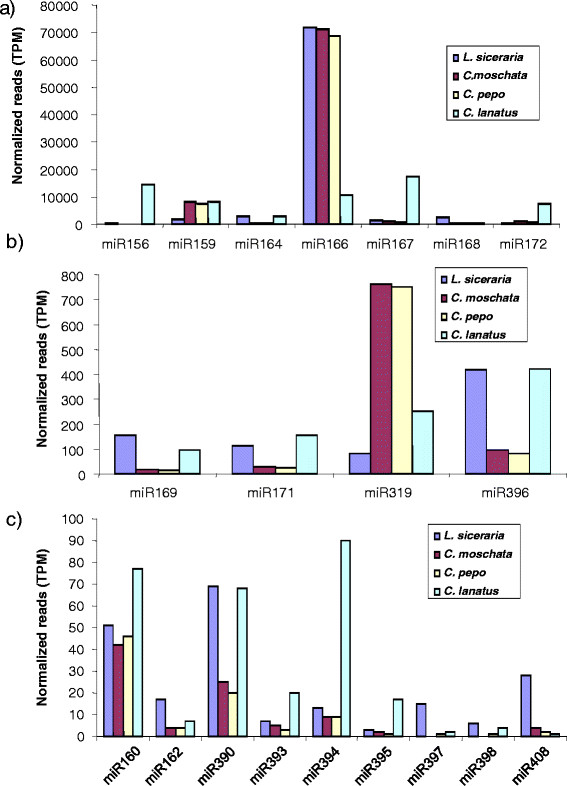
**Normalized miRNA profiles in 4 different cucurbits a). miRNAs that are abundantly expressed.** b). miRNAs with moderate abundance. c) miRNAs with low abundance.

As we employed deep sequencing approach to recover small RNA reads, we used read frequency to measure their abundance. Based on normalized read count per million (transcript per million, tpm), conserved miRNAs are divided into 3 groups: abundantly expressed (with read counts greater than 1000 tpm) miR156, miR159, miR164, miR166, miR167, miR168 and miR172; moderately expressed (with read counts 100–1000 tpm) miR169, miR171, miR319 and miR396; and those with low expression (with read counts less than 100 tpm) miR160, miR162, miR390, miR393, miR394, miR395, miR397, miR398, miR399 and miR 408 (Figure 2 a-c). miR395, miR397, miR398, miR399 and miR408 represented by less than 10 normalized reads were the miRNAs with least abundance in all 4 cucurbits (Additional file 1). miR395 and miR399 are induced in sulfate and phosphate deprived conditions, respectively [39-41] whereas miR397, miR398 and miR408 are induced when copper levels are low [42,43]. Thus the overall low abundance of these miRNAs (miR395, miR397, miR398, miR399 and miR408) in normal conditions is expected.

Many conserved miRNA families have multiple loci and the members often varied by one or two nucleotides. Most miRNA families were represented by multiple members; miR156 (5 members), miR159 (7 members), miR164 (6 members), miR165/miR166 (13 members), miR167 (7 members), miR169 (9 members), miR170/171 (11 members), miR172 (9 members), miR396 (7 members) and miR319 (6 members) (Additional file 1). However, 10 conserved miRNA families, miR160 (2 members, miR162 (1 member), miR168 (2 members), miR393 (2 members), miR390 (2 members), miR394 (1 member), miR395 (2 members), miR397 (1 member), miR398 (2 members) and miR408 (2 members) were represented by one or two members only. Overall, we recovered 83, 82, 82 and 81 miRNAs belonging to 22, 21, 22 and 29 miRNA families in bottle gourd, moschata, pepo and watermelon respectively. The highest number of miRNA families in watermelon is because of identification of less conserved miR824, miR827, miR858, miR1515, miR2916 and miR2950.

The number of miRNA variants within the miRNA families was similar in the most cases. However, we also observed preferential expression of specific members within the miRNA families. For instance, miR168 represented by two members (miR168a and 168b) that differ by one nucleotide and only one of these two loci preferentially expressed in all 4 cucurbit species (Additional file 1). This was previously shown both in Arabidopsis and rice [44,45]. In addition, we also found variation within the cucurbits in this study. For example, only one variant of miR396 was expressed at high level in watermelon (Additional file 1).

Few interesting observations emerged from the analysis of small RNA libraries are worth mentioning here. One such observation was the retrieval of miR158 homolog from watermelon but not from the other 3 cucurbits (Additional file 1). miR158 has been previously considered to be specific to Brassicaceae family [13]. In corroboration with our finding, miR158 was also identified in (*Cucumis melo*) small RNA libraries [28]. Another interesting observation was identification of miR894 homologs in these cucurbits. miR894 was reported from *Physcomitrella*[46], and in a few dicots such as sorghum [47] tomato [48] and cotton [49]. Although miR894 homologs were found in all four different cucurbits, watermelon has the highest frequency (Additional file 1). Identification of miR894 in all 4 cucurbits and its absence in several other dicots raises several interesting evolutionary questions including whether homologs of miR894 were selectively lost in certain lineages.

### Distinct miRNA profiles in four cucurbits

Normalized miRNA abundance indicated that several miRNA families (miR156, miR159 and miR160, miR164, miR167 and miR172) are expressed at high levels in watermelon compared to 3 other cucurbits. The differential abundance was strikingly high in case of miR156, where nearly 50 to 100 fold greater expression was noticed in watermelon as compared to 3 other cucurbits (Figure 2a). Similarly, expression of miR167 and miR172 varied considerably among the different cucurbits. Expression level of miR167 in watermelon was nearly 12-fold and 18-fold greater than in bottle gourd and *Cucurbita* genus, respectively (Figure 2a; Additional file 1). A similar trend was observed for miR172 (Figure 2a). In general, the miRNA abundance of most conserved miRNAs was the highest in watermelon and the least in bottle gourd (Additional file 1). However, a few miRNAs (miR162, miR168, miR397 and miR408) showed higher levels in bottle gourd compared to the 3 other cucurbit species studied here (Additional file 1). Of these, expression of miR168 in bottle gourd was nearly 10-fold higher when compared to moschata and pepo and 5-fold higher compared to watermelon (Figure 2a).

Overall, miRNA profiles are remarkably similar between moschata and pepo (Figure 2a; Additional file 1). Identical miRNA profiles between moschata and pepo is not surprising given the fact that these two belong to the same genus *Cucurbita*. Technically, this also confirms the uniform pooling of RNA samples from different tissues for library construction. The read count of miR166 family accounts for nearly 75% of the total reads, thus, is the most highly expressed in bottle gourd, moschata and pepo, whereas miR167 is the most highly expressed in watermelon. The second most abundant miRNA family was miR159 in moschata and pepo, miR164 in bottle gourd and miR156 in watermelon (Figure 2, Additional file 1). Low expression levels of miR162 and miR393 has been reported in diverse plant species [50,51]. Overall, our data demonstrated that considerable variabilty exists within the four species of Cucurbitaceae with regard to abundance of miRNA families.

### Dynamic regulation of conserved miRNAs in cucurbit tissues

**Figure 3:**
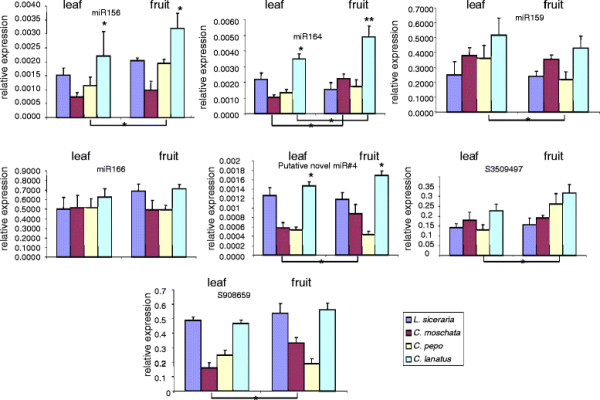
**Expression profiling of conserved and putative novel miRNAs in leaf and fruit tissues of different cucurbits by qPCR analysis.** The asterisks indicate that the expression values were significantly different. Asterisks with connecting line indicate differences in expression levels of leaf and fruit tissues in the same species that are significant (*: P <0.05; **: P < 0.01; Student’s *t* test).

**Figure 4:**
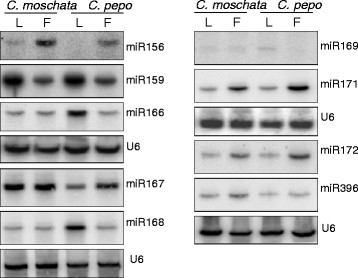
**Small RNA blot analysis in leaf and fruit tissues of *C. moschata *and *C. pepo*.** The U6 probe served as a loading control.

Since small RNA libraries were generated from the pooled equimolar RNA isolated from the leaf, stem and fruit tissues, it is difficult to ascertain if the differences in miRNA abundance observed in the libraries are due to similar/different abundance between these tissues. It is known that miRNA profiles are dynamically regulated in developing tomato fruit [48] suggesting an important role for the miRNAs in fruit development and maturation. Similarly, miRNA profiles in developing rice seeds are quite different [52] suggesting an important role for the miRNAs in fruit/seed development [53,54]. In order to identify miRNAs that are differentially expressed in fruit and leaves, we analyzed expression profiles of a subset of miRNAs (miR156, miR159, miR164 and miR166) in fruit and leaves of 4 cucurbits using q-PCR. miR164 expression levels in leaves as well as in fruits was significantly higher in watermelon when compared to the corresponding tissues of 3 other cucurbits as shown by q-PCR assay(Figure 3). miR164 expression levels were also significantly higher in fruits of moschata and watermelon compared to its levels in leaves (Figure 3). In general, the miR156 levels appear to be higher in fruit as compared to the leaf in all cucurbits (Figure 3). However, t-test indicated that the differences were only significant between leaves and fruits of pepo (Figure 3). On the other hand, miR159 levels seem to be higher in leaves as compared to fruit in all cucurbits, although statistically not significant (Figure 3). However, small RNA blot analysis in leaves and fruits of moschata and pepo indicated a greater abundance of miR159 in leaves compared to fruit tissue (Figure 4). Similarly, q-PCR analyses indicated no significant differences for miR166 levels between the leaves and fruits of different cucurbits (Figure 3), whereas small RNA blot comparisons between moschata and pepo showed a high level expression in leaves of pepo compared to the fruits. The discrepancy between q-PCR and small RNA blot analysis could be attributed to the fact that the small RNA blot analysis (hybridization-based determination) measures the abundance of entire miRNA family (most conserved miRNAs are represented by multiple members with the same sequence or sequence that differ by 1 or 2 nucleotides) whereas q-PCR is measuring one member of that particular family.

### Differential miRNA abundance in leaf and fruit tissues of moschata and pepo

*C. moschata* and *C. pepo* are two closely related species in Cucurbitacea family that show a high level of macro-synteny in their genomes [55]. In order to examine miRNA expression profiles in leaves and fruit tissues of these two species, we carried out blot analysis for several conserved miRNAs. Expression pattern of nine miRNAs analyzed in these two tissues had a similar profile in both species although some differences were noticed (Figure 4). For instance, miR166, miR167 and miR168 showed almost similar abundance in leaves and fruits of moschata, but their levels differed between leaves and fruits of pepo. miR166 and miR168 abundance was higher in leaves whereas miR167 abundance was higher in fruits of pepo. Expression level of miR159 was particularly high in leaves of both moschata and pepo. By contrast, miR156, miR171 and miR172 expression levels were relatively higher in fruits of both species (Figure 4). miR156 is generally considered to be an abundantly expressed miRNA in leaves. However, it has recently been noted that expression of miR156 increases during fruit ripening in tomato as observed here [56]. Abundance of miR396 was similar in leaves and fruits of pepo but slightly higher in fruits of moschata. Of the different miRNAs studied, miR169 had the least expression in both tissues and only a faint signal was detected, which is again consistent with the normalized read count (Figure 4 and Additional file 1). Expression level of miRNAs determined from q-PCR and small RNA blot analysis did not correlate in certain cases and such differences between different methodologies have been noticed earlier when measuring the miRNA abundance [57-59]. The observed differences could also be due to the differential sensitivity of each of these two methods’ i.e., q-PCR measures the abundance of individual miRNA, whereas small RNA blot analysis measures the abundance of multiple miRNAs belonging to a specific miRNA family.

### Putative novel miRNAs in cucurbits

**Figure 5:**
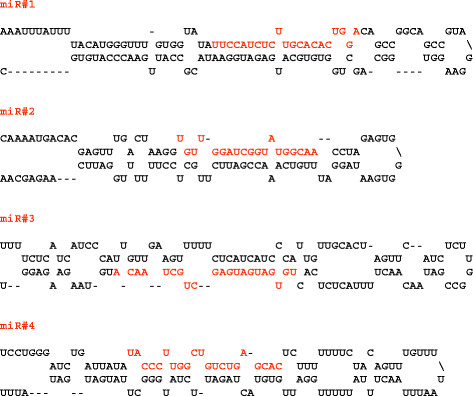
Predicted fold-back structures of putative novel miRNAs in cucurbits.

Our analysis of small RNA sequences in 4 cucurbit libraries has identified several potential novel miRNAs of 21 to 24 nt in length with characteristic fold-back structures of miRNA precursors (Figure 5). In general, novel miRNAs represent either lineage-specific or species-specific miRNAs and are expressed at low levels. Cloning of a miRNA^*^ in addition to the miRNA strand enhances the precision of miRNA annotation [8,60]. We did not detect any miRNA* corresponding to the potential new miRNAs in our libraries. However, the recovery of these sequences in several related species suggests their possible conservation and precise processing. Because we did not recover miRNA* species for these novel small RNAs, we annotate 4 small RNAs as potential new miRNAs in cucurbits (Table 2). Of these, two (miR#1 and miR#4) were recovered in all 4 cucurbits and two (miR#2 and miR#3) were recovered in 3 cucurbit species (bottle gourd, moschata and pepo), indicating that they might represent lineage-specific miRNAs. As observed for conserved miRNAs, the read numbers of novel candidate miRNAs in moschata and pepo were very similar. MicroRNAs that have been previously designated to be species-specific in other plants were also recovered in our libraries. For example, miR#1 that was recovered in all four cucurbit libraries was earlier shown to be specific to grapes (miRC2) [32]. With increasing sequencing depth, and analysis of diverse tissues, miRNAs previously identified as species-specific are becoming common in other plants. q-PCR analysis indicated that the miR#4 levels were significantly greater in leaves and fruits of watermelon compared to their respective tissues in 3 other cucurbits. miR#4 levels also displayed significantly higher abundance in fruits compared to leaves in moschata (Figure 3). Two other small RNAs s350949 and s908659 that were recovered in high numbers in our libraries, found to be derived from rRNAs representing rRNA-derived small RNAs. Interestingly these also showed differential expression between fruit and leaves (Figure 3).

**Table 2:** Normalized abundance (TPM) of putative novel miRNAs and their predicted targets in cucurbits

**miRNA**	**Sequence**	**Number of normalized reads**				**Targets**	
		**Bottle gourd**	**Moschata**	**Pepo**	**Water melon**	**Pepo**	**Watermelon**
miR#1	UUCCAUCUCUUGCACACUGGA	3	16	14	11	PU022623 (ubiquitin-protein ligase) ;	WMU58406 (acetolactate synthase)
						PU061865 (unknown protein) ;	WMU41511 (unknown protein)
						PU134802 (unknown protein)	
miR#2	UGUUGGAUCGGUAUGGCAA	13	17	13	0	PU119447 (unknown protein) ;	
						PU062878 (unknown protein) ;	
						PU005456 (zinc finger-like superfamily protein)	
						PU036654 (glycine rich protein);	
						PU014282 (unknown protein)	
miR#3	UGUGAUGAUGAGCUGCUAACA	7	1	2	0	PU057169 (glyceraldehyde-3-phosphate dehydrogenase)	
						PU043518 (unknown protein)	
miR#4	UACCCUUGGCUGUCUGAGCAC	19	26	23	3	PU084326 (cytochrome c oxidase)	WMU42945 (cytochrome c oxidase)

### Identification of TASi locus and tasiRNAs in cucurbits

Endogenous siRNAs comprise trans-acting siRNAs (tasiRNAs), natural anti-sense transcript-derived siRNAs (NATsiRNAs) and repeat-associated siRNAs (rasiRNAs) in plants [38]. Accurate annotation of siRNAs requires the knowledge of complete genomes. TasiRNAs are the only siRNAs that are conserved among diverse plant species and therefore these can be identified easily. Transcript from TAS3 locus harbors a tandem repeat of 21-nt almost identical sequences and possesses dual miR390 complimentary sequences. miR390 guided cleavage of TAS3 transcript at the 3′ end leads to production of dsRNA which is further processed by DCL4 to release tasiRNAs [61].

**Figure 6:**
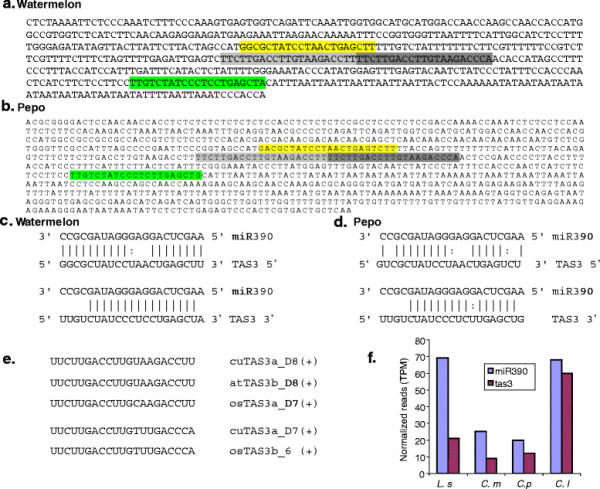
**Identification of ta-siRNA transcripts in cucurbits.** Nucleotide sequence of TAS3 locus derived transcripts in watermelon (**a**) and pepo (**b**). The 5′ and 3′ miR390 target sites are shown in yellow and green respectively and sequences that are complementary to auxin response factors are indicated in grey. Complementarity of TAS3 target sites and miR390 in (**c**) watermelon and (**d**) pepo. **e**). Sequence alignment of the processed TAS3 siRNAs in cucurbits to known TAS3 siRNAs from Arabidopsis and rice. **f**). Relative cloning frequency of TAS3a siRNAs and miR390 in four cucurbits: bottle gourd, moschata, pepo and watermelon.

In order to identify TAS3 siRNA loci in cucurbits and tasiRNAs derived from these loci, we analyzed small RNAs that are mapped to genome and surrounded by two miR390 target sites. At least one tasiRNA locus (TAS3a) with dual miR390 binding sites was identified in each of watermelon (unigene WMU79039) and pepo (unigene PU001490) genomes, and the two target sites are separated by 220 nt and 215 nt in watermelon and pepo respectively (Figure 6a,b). In both genomes, the 3′ target site has perfect complementarity at positions 9–12 nt from the 5′end of the miRNA, whereas the 5′ target has 2 mismatches and a G:U wobble in this position indicating that the 5′ target site may not be cleaved as shown in Arabidopsis (Figure 6c,d). Several siRNAs derived from these loci including tasiRNAs that aligned perfectly to tasiRNAs in Arabidopsis (atTAS3b_D8(+) and rice (osTAS3b_6(+) were found in the libraries (Figure 6e). In addition to these two precisely excised tasiRNA, several tasiRNA variants also appeared in the libraries (data not shown). Based on the predicted cleavage of TAS3 transcript by miR390, a positive correlation can be expected between miR390 and TAS3siRNAs and our normalized read counts agree with this view in all cucurbits analyzed (Figure 6f).

### miRNA targets in cucurbit transcriptome

In order to predict potential targets of miRNAs, currently annotated coding sequences from the cucurbit genomics database (http://www.icigi.org/) [62] were searched for the complimentary mRNA sequences. Further, EST databases in NCBI were also searched for transcripts that are potential targets of conserved miRNAs in cucurbits. Of the 4 cucurbits studied, very limited genomic information is available for *Lagenari*a and*. C. moschata* and therefore searching databases did not result in any hits. Accordingly, predicted targets were confined to watermelon (*C. lanatus var. lanatus*) and *C*. *pepo* genomes (Table 3). In addition, melon genome (*C. melo*) has also been searched for possible hits, because it is a closely related species of watermelon and genome has been partially sequenced. Because of the incomplete nature of genome sequence, unigenes in cucurbits were not fully annotated, but showed highest homology with that of known targets (Table 3). We were able to predict targets for several conserved miRNAs in our data set (miR156, miR159, miR164, 170/171, miR172, miR393, miR395, miR398). These targets include transcription factors (Squamosa Promoter-binding like factors, MYB transcription factors, Scarecrow-like/GRAS family transcription factors, Auxin Response factors) and genes implicated in auxin signaling (Transport Inhibition Response 1) and genes involved in sulfate assimilation such as ATP Sulfurylase 1. Target genes for conserved miRNAs are conserved across species and this trend was generally reflected in cucurbits. We also identified unigene sequences that are potential targets for the candidate novel miRNA sequences in watermelon and pepo genomes (Table 2, Additional file 2). Only one or two targets were identified for each of these candidate miRNAs, except for miR#2 for which multiple potential targets were found. These targets included transcripts encoding proteins involved in enzymatic activity and structural proteins although in many cases the targets were identified as unknown proteins or not annotated. With the availability of complete annotation of the sequence information in cucurbits, more accurate prediction and verification would become possible. Limited genomic information has hindered efficient prediction of targets for both conserved and novel miRNAs.

**Table 3:** Potential targets for conserved miRNAs in cucurbits

**miRNA**	**Watermelon**	**Pepo**	**Melon**	**Targets showing highest homology**
miR156	WMU3171	PU007476	MU34102	Squamosa promoter-binding protein-like (SPL) proteins
miR159	WMU2129	-	MU26436, MU24935,MU38257	MYB-like binding factors
	WMU63294			
miR160	-	-	MU38981	Auxin response factor (ARFs)
miR164	WMU579, WMU1219	PU030339	MU22717	NAC domain protein
	WMU1019			
miR170	-	-	MU2467	Scarecrow-like (SCL1) and GRAS family transcription factors
miR171	-	PU018031,PU001874, PU001874	MU25825	unknown proteins
miR319	WMU3608	PU002536,PU074309, PU067878,PU002536, PU115341	MU43136, MU27101	TCP/DNA binding proteins
miR393	WMU2032	PU077916	MU21869	Auxin receptors and BLH transcription factors
	-	-	MU38945	
miR395		PU044572,PU044572, PU044572	MU24817, MU27572	ATP sulfurylase
miR398	WMU38615	-	MU25081	Putative blue copper binding protein
miR408	WMU1327	-	MU25436	Plastocyanin-like domain-containing protein

## Conclusions

Comparative miRNA profiling in four cucurbits led to the identification of conserved (highly conserved as well as somewhat less conserved) miRNAs in cucurbits. Additionally, 4 putative new miRNAs are identified in these four cucurbits. Expression analysis showed differential regulation of several conserved miRNAs between leaves and fruit tissues of cucurbits. Our analysis also demonstrates considerable variability within four cucurbits with regards to expression of individual miRNAs. Even more strikingly, several miRNAs expression patterns differed between *C. moschata* and *C. pepo*, the two closely related cucurbits analyzed in this study. The predicted targets for conserved miRNAs suggested the involvement of miRNAs in regulating growth and development as well as other important physiological processes in cucurbits.

## Methods

### Small RNA library construction and sequence analysis

Total RNA was isolated from different tissues (leaf, stem and flesh, rind and placenta of the fruits) using TRIzol reagent and small RNA libraries were generated from four cucurbit species: bottle gourd (*Lagenaria siceraria* (accession Grif 1617 collection from India))*, Cucurbita moschata* (accession Grif 14244 Early Butternut) *Cucurbita pepo* (accession NSL98075 Table King), and watermelon *(Citrullus lanatus var. lanatus)* (PI 438676 Charleston Grey) by pooling equimolar amounts of total RNA from the aforesaid tissues. Construction of small RNA libraries from size fractionated RNA was carried out as described previously [13]. In brief, small RNA fractions of 18–28 nt were isolated from 15% denaturing polyacrylamide gels and sequentially ligated to 5′ and 3′ RNA adapters. Small RNAs ligated with adapters were converted to DNA by RT-PCR following Solexa protocol. The final PCR product was gel purified and sequenced by Genome Analyser II (Illumina).

Analysis of sequence reads from four cucurbit libraries was performed as described previously [13,16]. Briefly, adaptor sequences of the raw reads were removed and the small RNAs in between the adaptors were extracted. Unique small RNAs were obtained after eliminating redundant sequences. Reads mapped to known non-coding RNAs (rRNAs, tRNAs, snRNAs, snoRNAs) and repeats were removed from unique RNAs by aligning to databases Rfam [63] and Repbase [64], and the Plant Repeats database [65]. Remaining small RNAs were searched in the miRBase database [66] (release 16, obtained from http://microrna.sanger.ac.uk/sequences/ftp.shtml) to identify conserved microRNAs. Small RNAs mapping to known miRNAs of other plant species were designated conserved miRNAs in cucurbits. The genome sequence of cucumber was obtained from the Cucumber Genome DataBase [67]. To identify novel miRNAs, unique small RNAs with more than 10 genomic hits in cucumber genome were removed because they might possibly come from repeat-rich loci. The potential candidate miRNAs were identified by folding the flanking genome sequence of unique small RNAs using the RNAfold program [68]. To identify TAS genes and tasiRNAs in cucurbits, the genomes of watermelon and pepo genomes were searched using Hitsensor scores for possible miR390 binding sites [69] separated by 200 to 300-nt.

### Bioinformatic prediction of miRNA targets

To predict potential targets for cucurbit miRNAs, partially annotated coding sequences (unigenes) in watermelon, pepo and melon genomes were used for searching sequences complementary to the miRNAs (Cucurbit Genomics Database: http://www.icugi.org) [62]. A scoring matrix that allows a maximum of 3.5 mismatches (G:U accounts for half mismatch) between the miRNA and its target mRNA was used in the analysis [70]. In order to get putative annotation for the target genes, sequences identified as targets were BLAST searched and the genes showing highest homology in other plants were assigned as putative annotation in cucurbits.

### qRT-PCR analysis

Expression profile of miRNAs in cucurbits was verified by performing stem loop RT-PCR as described previously [71]. One microgram total RNA in leaf and fruit tissue was used to perform RT reaction with miRNA specific RT primers and 1:10 diluted cDNA was used in q-PCR experiments. RT stem-loop primers and all other primers used are given in Additional file 3. Real-Time PCR was carried out using Maxima^TM^ SYBR Green q-PCR Master Mix in an ABI7500 Real-Time PCR System. The relative expression was obtained using delta-Ct method and actin as reference gene. The data shown was mean of 3 replicates. Student t-test was used to determine the significant differences between different tissues as well as in the same tissue of different cucurbits.

### Small RNA blot analysis

Total RNA (100 μg) was resolved on a 15% polyacrylamide gel containing 7 M urea in TBE buffer. Size-fractionated small RNAs were then transferred to Hybond-N + (Amersham) membranes, UV cross-linked and baked for 1 h at 80°C. After pre-hybridization, blots were hybridized at 38°C with a ^32^P-labeled DNA oligo probe complementary to miRNA sequence. After washing, blots were exposed to phosphor screen and scanned using a phosphorimager. Blots were used for reprobing after thorough stripping.

## Competing interests

The authors declare that they have no competing interests.

## Authors’ contributions

GJ constructed the small RNA library, YZ analyzed the libraries, NP isolated the RNA from different tissues, GJ performed the expression analysis with KGs’ assistance, RS and UKR designed the study, GJ and RS wrote the manuscript. All authors read and approve the manuscript.

## Supplementary Material

Additional file 1 **Table 1.** Identified conserved miRNAs from four different *Cucurbitaceae* members. Click here for file

Additional file 2 Target prediction for new miRNAs. Click here for file

Additional file 3 **Table 2.** Primers used for real-time RT PCR analysis. Click here for file
