# Supplementary material for: Characterization of the small RNA component of leaves and fruits from four different cucurbit species
Source: BMC Genomics. 2012 Jul 23;13:329. doi: 10.1186/1471-2164-13-329 (PMC3431224; doi:10.1186/1471-2164-13-329)
Supplement: Additional file 1 — Table 1. Identified conserved miRNAs from four different Cucurbitaceae members. [file 1471-2164-13-329-S1.doc]

Suppl. Table 1. Identified conserved miRNAs from four different *Cucurbitaceae* members

| miRNA | sequence | bottle gourd | moschata | pepo | water  melon |
| --- | --- | --- | --- | --- | --- |
| MIR156 | UGACAGAAGAGAGGGAGCAC | 0 | 1 | 1 | 37 |
| MIR156 | UGACAGAAGAGAGUGAGCAC | 90 | 35 | 34 | 5217 |
| MIR156 | UUGACAGAAGAGAGAGAGCAC | 7 | 0 | 0 | 207 |
| MIR156 | UUGACAGAAGAUAGAGAGCAC | 74 | 25 | 21 | 3698 |
| MIR156 | UGACAGAAGAGAGUGAGCACA | 89 | 34 | 33 | 5236 |
| MIR158 | UCCCAAAUGUAGACAAAGCA | 0 | 0 | 0 | 7 |
| MIR159 | UUUGGAUUGAAGGGAGCUCUA | 382 | 1686 | 1555 | 4919 |
| MIR159 | CUUGGAUUGAAGGGAGCUCUA | 334 | 1518 | 1389 | 86 |
| MIR159 | UUUGGACUGAAGGGAGCUCUA | 1 | 6 | 6 | 0 |
| MIR159 | UUGGAUUGAAGGGAGCUCCA | 328 | 1519 | 1398 | 0 |
| MIR159 | UUUGGAUUGAAGGGAGCUCUG | 364 | 1640 | 1513 | 1358 |
| MIR159 | UUUGGAUUGAAGGGAGCUCUU | 358 | 1627 | 1493 | 1385 |
| MIR159 | UUUGGAUUGAAGGGAGCUCCU | 0 | 0 | 0 | 304 |
| MIR160 | UGCCUGGCUCCCUGUAUGCCA | 27 | 22 | 24 | 76 |
| MIR160 | UGCCUGGCUCCCUGUAUGCCG | 24 | 20 | 22 | 1 |
| MIR162 | UCGAUAAACCUCUGCAUCCAG | 17 | 4 | 4 | 7 |
| MIR164 | UGGAGAAGCAGGGCACAUGCU | 1 | 0 | 0 | 1 |
| MIR164 | UGGAGAAGCAGGGUACGUGCA | 1 | 0 | 0 | 1 |
| MIR164 | UGGAGAAGCAGGGCACGUGCA | 875 | 154 | 147 | 1273 |
| MIR164 | UGGAGAAGCAGGGCACGUGCU | 883 | 156 | 148 | 1607 |
| MIR164 | UGGAGAAGCAGGGCACGUGCG | 874 | 154 | 146 | 58 |
| miR164 | UGGAGAGGCAGGGCACAUGCU | 36 | 2 | 1 | 0 |
| MIR165 | UCGGACCAGGCUUCAUCCCCC | 8 | 12 | 10 | 6 |
| MIR166 | UCGAACCAGGCUUCAUUCCCC | 6 | 5 | 8 | 4 |
| MIR166 | UCGGACCAGGCUUCAUUCCCUU | 6203 | 5753 | 5571 | 210 |
| MIR166 | UCCGGACCAGGCUUCAUUCCC | 6645 | 5994 | 5788 | 2 |
| MIR166 | UCGGACCAGGCUUCAUUCCCCC | 6889 | 6276 | 6060 | 4933 |
| MIR166 | UCGGACCAGGCUUCAUUCCUA | 8156 | 8329 | 8083 | 72 |
| MIR166 | UCGGACCAGGCUUCAUUCCUC | 8156 | 8330 | 8084 | 80 |
| MIR166 | UCGGACCAGGCUUCAUUCCCU | 8859 | 8829 | 8547 | 0 |
| MIR166 | UCGGACCAGGCUUCAUUCCCC | 8963 | 8965 | 8694 | 4931 |
| MIR166 | UCGGACCAGGCUUCAUUCCC | 9147 | 9422 | 9128 | 0 |
| MIR166 | UCGGACCAGGCUUCAAUCCCU | 4 | 5 | 3 | 0 |
| MIR166 | UCGGACCAGGCUUCAUUCCUU | 8751 | 9076 | 8781 | 72 |
| MIR166 | UCGGACCAGGCUUCAUUCCCGU | 0 | 0 | 0 | 140 |
| MIR167 | UGAAGCUGCCAGCAUGAUCUG | 201 | 115 | 111 | 4975 |
| MIR167 | UGAAGCUGCCAGCAUGAUCUGG | 178 | 110 | 106 | 4978 |
| MIR167 | UGAAGCUGCCAGCAUGAUCUGA | 180 | 112 | 108 | 5085 |
| MIR167 | UGAAGCUGCCAGCAUGAUCUU | 200 | 112 | 107 | 686 |
| MIR167 | UGAAGCUGCCAGCAUGAUCUAA | 286 | 229 | 207 | 818 |
| MIR167 | UGAAGCUGCCAGCAUGAUCUA | 208 | 150 | 130 | 538 |
| MIR167 | UGAAGCUGCCAGCAUGAUCUC | 198 | 111 | 106 | 193 |
| MIR168 | UCGCUUGGUGCAGGUCGGGAA | 2470 | 255 | 240 | 476 |
| MIR168 | UCGCUUGGUGCAGAUCGGGAC | 8 | 5 | 6 | 0 |
| MIR169 | AAGCCAAGGAUGACUUGCCGG | 1 | 1 | 1 | 1 |
| MIR169 | AAGCCAAGGAUGAAUUGCCGG | 70 | 6 | 4 | 68 |
| MIR169 | UAGCCAAGGAUGAAUUGCCGG | 67 | 5 | 4 | 2 |
| MIR169 | UAGCCAAGAAUGACUUGCCUA | 12 | 0 | 0 | 0 |
| MIR169 | UAGCCAAGGAUGACUUGCCUG | 0 | 2 | 2 | 3 |
| MIR169 | UAGCCAAGGAUGACUUGCCUGC | 0 | 2 | 2 | 3 |
| MIR169 | UGAGCCAAGGAUGACUUGCCG | 1 | 1 | 1 | 1 |
| MIR169 | CAGCCAAGGAUGACUUGCCGG | 1 | 1 | 1 | 16 |
| MIR169 | UGAGCCAAGAAUGACUUGCCGGC | 4 | 1 | 0 | 1 |
| MIR170 | UGAUUGAGCCGUGUCAAUAUC | 0 | 2 | 2 | 0 |
| MIR171 | UUGAGCCGCGUCAAUAUCUCC | 1 | 0 | 0 | 0 |
| MIR171 | UUGAGCCGCGCCAAUAUCACU | 1 | 0 | 0 | 0 |
| MIR171 | UGAGCCGUGCCAAUAUCACGA | 0 | 1 | 1 | 0 |
| MIR171 | UUGAGCCGUGCCAAUAUCACA | 0 | 1 | 1 | 0 |
| MIR171 | UUGAGCCGUGCCAAUAUCACG | 0 | 1 | 1 | 0 |
| MIR171 | UGAUUGAGCCGUGCCAAUAUU | 22 | 1 | 0 | 3 |
| MIR171 | UGAUUGAGCCGUGCCAAUAUC | 22 | 1 | 1 | 131 |
| MIR171 | AGAUUGAGCCGCGCCAAUAUC | 23 | 11 | 10 | 0 |
| MIR171 | UGAUUGAGCCGCGCCAAUAUCU | 24 | 12 | 10 | 20 |
| MIR171 | UUGAGCCGUGCCAAUAUCAC | 20 | 2 | 2 | 0 |
| MIR172 | UGAGAAUCUUGAUGAUGCUGCAU | 0 | 0 | 0 | 7060 |
| MIR172 | AGAAUCUUGAUGAUGCUGCAU | 44 | 195 | 171 | 0 |
| MIR172 | GGAAUCUUGAUGAUGCUGCAG | 44 | 190 | 166 | 0 |
| MIR172 | AGAAUCUUGAUGAUGCUGCAG | 44 | 194 | 171 | 68 |
| MIR172 | AGAAUCUUGAUGAUGCUGCA | 45 | 198 | 173 | 0 |
| MIR172 | GGAAUCUUGAUGAUGCUGCAU | 44 | 191 | 167 | 267 |
| MIR172 | UGGAAUCUUGAUGAUGCUGCAG | 0 | 0 | 0 | 16 |
| MIR172 | GGAAUCUUGAUGAUGCUGCAGCA | | 0 | 0 | 16 |
| MIR172 | UGAAUCUUGAUGAUGCUGCAC | 0 | 0 | 0 | 1 |
| MIR319 | UUGGACUGAAGGGAGCUCCCA | 13 | 125 | 123 | 62 |
| MIR319 | CUUGGACUGAAGGGAGCUCCC | 13 | 127 | 126 | 71 |
| MIR319 | UUGGACUGAAGGGAGCUCCUU | 12 | 113 | 109 | 0 |
| MIR319 | UUGGACUGAAGGGAGCUCCCU | 14 | 129 | 128 | 109 |
| MIR319 | UUGGACUGAAGGGAGCUCCC | 18 | 139 | 138 | 0 |
| MIR319 | UUGGACUGAAGGGAGCUCCU | 13 | 127 | 125 | 10 |
| MIR390 | AAGCUCAGGAGGGAUAGCGCC | 36 | 13 | 10 | 60 |
| MIR390 | GAGCUCAGGAGGGAUAGCGCC | 33 | 12 | 10 | 8 |
| MIR393 | UCCAAAGGGAUCGCAUUGAUCU | 3 | 2 | 3 | 6 |
| MIR393 | UCCAAAGGGAUCGCAUUGAUCC | 4 | 3 | 3 | 14 |
| MIR394 | UUGGCAUUCUGUCCACCUCCAU | 13 | 9 | 9 | 90 |
| MIR395 | CUGAAGUGUUUGGGGGAACUC | 3 | 2 | 1 | 8 |
| MIR395 | UUGAAGUGUUUGGGGGAACUC | 0 | 0 | 0 | 9 |
| MIR396 | UUCCACAGCUUUCUUGAACUGU | 0 | 0 | 0 | 300 |
| MIR396 | UUCCACAGCUUUCUUGAACUG | 122 | 18 | 16 | 0 |
| MIR396 | GUUCAAUAAAGCUGUGGGAAA | 28 | 7 | 6 | 0 |
| MIR396 | UUCCACGGCUUUCUUGAACUU | 12 | 17 | 14 | 3 |
| MIR396 | UUCCACAGCUUUCUUGAACUU | 125 | 18 | 17 | 67 |
| MIR396 | UUCCACAGCUUUCUUGAACUA | 120 | 16 | 15 | 0 |
| MIR396 | UUCCACGGCUUUCUUGAACUG | 12 | 18 | 15 | 50 |
| MIR397 | UCAUUGAGUGCAGCGUUGAUG | 15 | 0 | 1 | 2 |
| MIR398 | UGUGUUCUCAGGUCACCCCUG | 0 | 0 | 1 | 4 |
| MIR398 | UGUGUUCUCAGGUCGCCCCUG | 6 | 0 | 0 | 0 |
| MIR408 | UGCACUGCCUCUUCCCUGGCUG | 26 | 2 | 1 | 0 |
| MIR408 | AUGCACUGCCUCUUCCCUGGC | 2 | 2 | 1 | 1 |
| MIR894 | CGUUUCACGUCGGGUUCACC | 85 | 81 | 92 | 35 |
| MIR824 | UAGACCAUUUGUGAGAAGGGA | 0 | 0 | 0 | 1 |
| MIR827 | UUAGAUGACCAUCAACGAAAA | 0 | 0 | 0 | 1 |
| MIR858 | UUUCGUUGUCUGUUCGACCUU | 0 | 0 | 0 | 1 |
| MIR1515 | UCAUUUUUGCGUGCAAUGAUCC | 0 | 0 | 0 | 4 |
| miR2111 | UAAUCUGCAUCCUGAGGUUUA | 7 | 4 | 5 | 60 |
| MIR2111 | UAAUCUGCAUCCUGAGGUCUA | 0 | 0 | 0 | 1 |
| MIR2916 | UGGGGACUCGAAGACGAUCAUAU | 0 | 0 | 0 | 1 |
| MIR2950 | UUCCAUCUCUUGCACACUGGA | 0 | 0 | 0 | 11 |

Supp Table 2: primers used for real-time RT PCR analysis

| cuc-miR156-RT | GTCGTATCCAGTGCAGGGTCCGAGGTATTCGCACTGGATACGACGTGCTC |
| --- | --- |
| cuc-miR159-RT | GTCGTATCCAGTGCAGGGTCCGAGGTATTCGCACTGGATACGACTAGAGC |
| cuc-miR164-RT | GTCGTATCCAGTGCAGGGTCCGAGGTATTCGCACTGGATACGACAGCACG |
| cuc-miR166-RT | GTCGTATCCAGTGCAGGGTCCGAGGTATTCGCACTGGATACGACGGGGAA |
| cuc-miR171-RT | GTCGTATCCAGTGCAGGGTCCGAGGTATTCGCACTGGATACGACGATATT |
| cuc-miR156-FP | GCGGCGGTGACAGAAGAGAGT |
| cuc-miR159-FP | CGGCGGTTTGGATTGAAGGGA |
| cuc-miR164-FP | CGGTGGAGAAGCAGGGCA |
| cuc-miR166-FP | TCGCTTCGGACCAGGCTTCA |
| cuc-miR171-FP | TTCCTTGATTGAGCCGCGCC |
| cuc-novel 1( s6657814) RT | GTC GTA TCC AGT GCA GGG TCC GAG GTA TTC GCA CTG GAT ACG ACG TGC TC |
| cuc-novel 9(s3509497) RT | GTC GTA TCC AGT GCA GGG TCC GAG GTA TTC GCA CTG GAT ACG ACA GCC GA |
| cuc-novel 10 (s908659) RT | GTC GTA TCC AGT GCA GGG TCC GAG GTA TTC GCA CTG GAT ACG ACC TGC CA |
| cuc-novel 1(s6657814) FP | GGC GGT ACC CTT GGC TGT CT |
| cuc-novel 9( s3509497) FP | GCG GCG GCC CAG TCC CGA ACC CG |
| cuc-novel 10 (s908659) FP | GCG GCG GAC AGG GTA TTG TAA G |
