# Supplementary material for: Characterization of the small RNA component of leaves and fruits from four different cucurbit species
Source: BMC Genomics. 2012 Jul 23;13:329. doi: 10.1186/1471-2164-13-329 (PMC3431224; doi:10.1186/1471-2164-13-329)
Supplement: Additional file 2 — Target prediction for new miRNAs. [file 1471-2164-13-329-S2.doc]

**Additional file 2**

**Target prediction for new miRNAs**

***1. Target for cuc-miR1 (*sequence *UUCCAUCUCUUGCACACUGGA)***

***a)* watermelon**

**WMU58406 sequence (acetolactate synthase)**

GGTTCAAGAAATAAAATGTCAGCATTAATCGTATAGCTGGAGTTTTTGCTAGAAGAGGATATAACATTGAATCACTTGCTGTTGGCCTGAACAAGGACAAGGCTCTTTTCACTATAGTTGTCTCCGGGACTGAAAAGGTGTTAGAACAAGTTATTGAGCAGCTCCACAAGCTTGTGAATGTTCTGAAGGTTGACGATCTCTCCAGTGTGCAACAAGTGGAACGTGAACTGATGCTTATAAAAATAAATGCTGAGCCGAACATATCGTGCTGAGATTAAGAGGTTAGTGGACATCTTCAGAGCGAAAATTGTAGATATCCCAGATGATTC

**WMU58406 alignement**(201-221)

WMU58406 5'UCCAGUGUGCAACAAGUGGAA 3'

||||||||||||x|x:|||||

cuc-miR1 3'AGGUCACACGUUCUCUACCUU 5'

**WMU41511 sequence (hypothetical protein)**

GTCAATCGAAAGAGCCGTCAGTAGAAAGAGTACGAGGGTTTTAGAGAATCGACTCCTAATTTCAGTGTTCAAGAGATAGAGAGAGTGAAGAAGAATTAGGGTTTTAGACAAAATGGCAATGGGACTAAGGCCTTACACGGAGAGAACAGGAAATGGCTTGGGATTACCAGCTTTCGACGCAAATCAAGGCGAGGTTCTCATGCACATAATGCCCGCCGTCGGCATCGTGTTTGATAATCGGCCTCCCGAGTTTCCTGGCACTCTCTACATATCCTCCAGGCAGGTGGTTTGGTTGAGTGATGTAGACATGGCCAAAGGTTATGCAGTTGATTTTCTTGTCAATGTCATTGCATGCAGTTTCAACAGACCCAGAGGCCTATTCATCTCCTTGTCTGTATATTCAGATTGATACTGGAGATGATGAAGAGTCACATAATTCAGATTCAGAATGCCACGGTGAAGAGTCTGAGAATTTAGTGTTAGCTAAAACGTAGAATTTATCTCGGGATTTGACTTTCGTTTACTAA

**WMU41511 alignment**(61-81)

WMU41511 5'UUCAGUGUUCAAGAGAUAGAG 3'

|:||||||x||||||||x||:

cuc-miR1 3'AGGUCACACGUUCUCUACCUU 5'

***b)* pepo:**

**PU022623 sequence (ubiquitin-protein ligase)**

AGCAACTTCTCTTTTCGGAGTAAAGTCTCAATCAAGACACTCTGGAAAGGGTGCTTTCGAGGCTACCGACCTCGGCATTCTTTCGTCTTTCTTCAGTGTGCAAAAGATGGAAATCAGTTGCAGCTTCTTCAAGTTTCAGGTCTGCTTGCTCAGACTATTTCGGCACGCGACCCCTGGTTTTTCATGGTCGATTCACATCTCAACCGCTCGATTGTTTTTCGACTCGACTGAGAAAAATTGGAAGAAACTTAACTATCCACACCTCCTTCAGAATCGTCATCTCGATTCGATCCCCGTTGCAGCCTCTGGTGGTTTGATTTGCTTTCGCAATTCGGAGGGAAACTTCATAGTGACCAATCCTTTAACAGGCTCTTGTACGTGAGCTTCCTCCTGTGGATCTTGACCAGAAAAGCAATCTCTTCTTGCCTATTGTGATGAATG

PU022623 alignment(92-112)

PU022623 5'UUCAGUGUGCAAAAGAUGGAA 3'

|:||||||||||x||||||||

cuc-miR1 3'AGGUCACACGUUCUCUACCUU 5'

**PU061865** **sequence (unknown protein)**

AAGATTAGTTTTACAACTTGAAGGGAAAACCGAAGCAAATGGTTCTGCAGTGGCTCAAGTGCTATTCATAAAGTCAAGAAATAGAAGTAGGTGTACGTTCATTTTGTTGTCAGTTCAAGTTATTGCTGAAGACTGAAGAATTCAATCAGCATAGTGTTACTGGTGATTACTTAGGTTCAGAAACACTTAGATATGCAAAATGTGTATAATTCAGTGCAGTAGCTTCTGCTGGACAGAGATAACAACAGAGCTGCAGGTTATAGCAATTCCCTCGCTTGATGCCCTTCCTGGAGCTAGTAGCCAGAGGGCGGGTCAGGTTGTAATACTGCTGGTGTGAGAATGAAAGATGCCATTTACTTTATTCAGTGTGCAAGAGATTGAAATTAGGGCAAGCATCCAAGAAAGGTGGATTGAGGGGTTTCCAGCTAAATCACAAGAAAGCTAATATCTCTAACTTGTTATATTCTTGAGAAGTACCATGAATTATCTTTAGG

PU061865 alignment(362-382)

PU061865 5'UUCAGUGUGCAAGAGAUUGAA 3'

|:|||||||||||||||x|||

cuc-miR1 3'AGGUCACACGUUCUCUACCUU 5'

**PU134802 sequence (unkown protein)**

ACGCGGGAACCTTCATTTCAAATGGATAGGCACAAGTTTTGTTTTGATTTAATTAGGGGTAAAAAAATTATGATAGGCCCTACGTTTAACTTTATTATATTGATCAATTAGCTGTGTTCCTGACAAAATCTGACATATCTTCTTTCTTCTCAGTTACTTTATTCAGTGTGCAAGAGATTGAAATTAGGGCAAGCATCCAAGAAAGGTGGATTGAGGGGTTTTCCAGTTAAATCACAAGAAAGCTAATATCTCTATACCTTGTTATATTCTTGAGAAGTAACCATGAATTATCTTTAGGTACAGGAAAGGATAGAAATAGGACTAACCTCAGGGCTAGTGTCTTTTCTACTCCTTTCTTTAACTAAGGAGTGAAGAGAAGTTAGATTTC

PU134802 alignment(162-182)

PU134802 5'UUCAGUGUGCAAGAGAUUGAA 3'

|:|||||||||||||||x|||

cuc-miR1 3'AGGUCACACGUUCUCUACCUU 5'

----------------------------------------------------------------------

***2. Target for* *cuc-miR2* (sequence UGUUGGAUCGGUAUGGCAA)**

**a) pepo**

**PU119447 sequence (unknown protein)**

ACGCGGGGGAGAAGACGAAGATTCATTCGCATTTGCCATACCGGGCCAGCAATTCCGCCACCAACTGCAACCACAAGGCTTTTTTGGTAGGAAACTTAGAATTTTCCGATCCTCCTCCGGTGCCCTCTGATAATCTCCCCGATGGTTCAAAACTCTCCACAACCGCCGCCACTCGAGGAACACACCACACGAAACCGACCCGACGAACCGAACTTAGTCCGAAACTACTTCCCTAA

PU119447 alignment(33-51)

PU119447 5'UUGCCAUACCGGGCCAGCA 3'

|||||||||||:x|||:||

cuc-miR2 3'AACGGUAUGGCUAGGUUGU 5'

**PU062878 sequence (unknown protein)**

TCGATCCGTTCCCTCCTCTCAGTGGCATCATTAGAGGCACCCCTTTGCTTGCCCATTTGTAAATTGAGAAATGGAATTGAAACTGACCACCACCTATTTTAATCTTCTACACTATCTTCTCCCTTCGTTGAACTGTTCCTATTCGACGCATTATCGGACTTTCTGAAGGATGGCATCTCGGCGCTGCTAAGAGGTTGGAATTCAGATGACAGAATGCCAGGCCGATCCAACAAATTGTGATCACTTTTAAGATCCTCGAAACGATGGCTCGAAGTGCTGGAAGAAAATCTAGACAGAGTGAACCTAGAAGCATTTGTATGGCTTCCATTTGACACAACAACCTTGTTTCTTAAGGGGCTCGAATTGTTCGACCCCAACGCTGGCAGATCAGTCCCTTTAGCCAATCTAGATGGTAGGCTTAATTCTGCTGGGAGGGAAGAACTTCCGAATGGCTCTGCCGGCGGCGGAAGTTGCCTAGCAGGACTTGAGACCCT

**PU062878 alignment mRNA (214-232)**

PU062878 5'AUGCCAGGCCGAUCCAACA 3'

x|||||x:|||||||||||

cuc-miR2 3'AACGGUAUGGCUAGGUUGU 5'

**PU005456 sequence (zinc finger-like superfamily protein)**

TGGACCCATGGTGCCAGGGCCCATTGGCTAAAGTCCTATTGCCATAGCGATCCATTATGGGTGGCATACCATACACTGCACTATTTCCCATCATAGATCCACTTGAATAAGGTGGAGGCCCAGATAGGTGCAAGGGGTCTATAGGGACTGCCTGCAGAAAACCGGCTGCCGTAGTTGTAATGATATGCTGATCCCCCAGAAAATGGCACATCATAGGGAGGTATAGATGAACCGTTGAATAGTGGAAGAACCATAGTGGTGGTAACACCTAGGATACATTGAAGAGGGTACACCTGAAACCCAATGTAAGGGGCAGAATAAGAGTAACCCTGAAGTGCTTGAACGGGCTTGGCAGCAGATTTAGAGTTATGATCAGCAGGACGTGGCTGAGTGCAATTGCGCATATTGCAAGTTGTTCTAAACGAAAATTAGACATTTCCACAACTAGGGCAAGTACCAATCATCTTCCCTTCCGGCTACCACTCACGTTCTAGCAC

PU005456 alignment(39-57)

PU005456 5'UUGCCAUAGCGAUCCAUUA 3'

||||||||x|||||||x:|

cuc-miR2 3'AACGGUAUGGCUAGGUUGU 5'

**PU036654 sequence(glycine rich protein)**

TGAACCAGTACTAGACGATTTCAAGAGAATAAAGGGTCACATAGATCTCGATTCCCAACCCAAGAAACCCTCCGGTCGGGACTTCAAGGAGATCGCCGGGAGTTTCTGGTCAGTCGCTTCTGTTTTCAGCAAGAAATGGCAGAAATGGAGAGATAAGCAGAAGCTCAAGAAGAGCAGAAATGGCGGTGGCTCCGCCACATTGCCAGTGGAGAAGCCGATCGGACGTCAATTCAGAGAAACACAGTCGGAGATAGCCGATTACGGCTATGGCCGTCGGTCCTGCGATACCGATCCAAGATTCTCCCTCGACGCCGGCCGAATGTCCTTCGACGATCCTCGTTACTCCTTCGATGAACCACGAGCTTCTTGGGATGGCTATTTAGTAAGCCGTACTTTCACCAGAATGCCGACGATGCTCTCTGTTGTTGAAGATGCTCCGATTCATGTTTCTCGAACGGATACTCAAATTCCGGTCGAAGAACCTGCAATTTCCGGCAATGACGATGAAACCGTCCCCGGTGGTTCGGGAACAAACCAGAGATTACTACTCCGACTCCTCTCGCCGAAGAAGAAGCCTCGACAGATCAAGCTCACTAAGGAAGACGGCAGCGGCA

PU036654 alignment(280-298)

PU036654 5'CUGCGAUACCGAUCCAAGA 3'

x|||x||||||||||||x|

cuc-miR2 3'AACGGUAUGGCUAGGUUGU 5'

**PU014282 sequence (unknown protein)**

ACGTCGGGTTATGTGGTTAAAGCTGTATGCTATGTTAAAAAAACATGGAATATACAAAATTTACCATACCAATCCAATGTCAATGGTATTTCTTTTGCTTTTTTTCTTCACATAGTTATGAAGGTGGCGCCTACGCTTTTTGGTCATATGCAAACCATTTGTCACATAGCTTTGAATTCTGAGAACAATTTGTCGAAATAGAGCTTTCAGATCTTGTGTTTTGTGTAGTTTATGGCAGAATAATATGAACATGTAAGTCAACCTATTCAGACTTGGAAGTGAAGAATATATGCCAAATATTGCTGACCTACTAAATAAAGTAACATAGTTGAGAGGTTCAGTCAGTTTATATCTG

PU014282 alignment(61-79)

PU01428 5'UUACCAUACCAAUCCAAUG 3'

||x|||||||x||||||::

cuc-miR2 3'AACGGUAUGGCUAGGUUGU 5'

***3. Target for cuc-miR3* (sequence UGUGAUGAUGAGCUGCUAACA)**

a**) Targets in pepo**

**PU057169 sequence (glyceraldehyde-3-phosphate dehydrogenase)**

ACGTCGGGGGTAGTATTTGAGGAGCGGAACCTTGAGGCGGCGCTACTGACAAGAGTGGTCACGATCAGCTCGGAGAAGGTACGGCCAGGTTAGAAGCTCATCATCAGAAGGCATTACAACAAATTCTCAAATAACCAGAGGAAATTCGGCGTCCATGGGCAAGAAGATGAAGATCATCCCCGTGCTTCTGATCGGCTGCGGCGGTGTTGGCCGTCAGCTCCTCCACCACATCGTATCCTGCCGATCACTTCACGCTAAACAGGGCGTCCACTTGCGAGTTATAGGAGTATGTGATAGTAGATCACTAGTCGTTGCATCTGATGCGCTCACAACCGAGTTAAATGACAAGATTTTAATGGAAATTTGTCATGTCAAGTCGAGTGGTGGCTCTTTGTCAAATCTTAGCAATTTGGGTGAGAGCAAACTATTTTCCGATACAGAAGCAACTAAAAAGTTATTGATATTGCGACTCTTCTCGGTAAAGCGACAGGTCTGGCCTGTGTTGATTGCTCTGCCAGTTCTGACACTGTTAATGTGTTAAAGCAAGTGGTTGACTTGGGTTGTTGCATTGTTTTGGCAAATAAGAAGCCTTTAACCTCTACAATTGAGGATTATAACAAACTGACTCCACATCCACGTCGTATTCGAC

PU057169 alignment(88-108)

PU057169 5'GGUUAGAAGCUCAUCAUCAGA 3'

x|||||x||||||||||||x|

cuc-miR3 3'ACAAUCGUCGAGUAGUAGUGU 5'

**PU043518 sequence (unknown protein)**

TAACTGAGTCGCAAATTACAGCTGTTGTAAACGTAACGTACGCTAACTTCAACCATCATATTCTACAAAAAATATCTCAGCTTTTGGACCCAAAAACCATCCACATTCCAGCAATGATGATCTCTGAGAACAAGTTAACGACCATGACACAGGAATACTGTGACTTAGTTGGAATGGTAGTCCCTTTATCAAAGCTAAACTTTAGGACTTTGTTCGTCTGAAGGATGTTCAGGCGAAGCAGGTAGTTGTTCGTTTCCGTCGGTTTCAGCTAAAACAGGATCGTTGGGAGGTTCCTCTCCACTATCATTAGTCTCTGGGTAAAAATTAATGGGAAACTTTGTAACCCTCGGTTTATCAGAGAGTAATGTTTCTCTGAACCCTATCGATCAGGACTTTGGCTGGAGGTTCTTCTCTTAGCTGCTCATCGTCATAATCATTGTTTATGTAGTAACCGACGCGGACAAACTCCTGACCCATGTATGAGCAGGTCAACAGAAGTACAGTTACACCAATGATATCTTCTTCAGGAATTCTGGACGGGTCCGGAGGGTCAGCCTGCAATACGAAA

PU043518 alignment(412-432)

PU043518 5'UCUUAGCUGCUCAUCGUCAUA 3'

|x|||||x|||||||:|||:|

cuc-miR3 3'ACAAUCGUCGAGUAGUAGUGU 5'

-----------------------------------------------------------------------

***4.* Target for *cuc-miR4* (sequence UACCCUUGGCUGUCUGAGCAC)**

**a) Target in watermelon**

**WMU42945 sequence (cytochrome c oxidase)**

GGGGTTCAACTTAAGCTGCGTCCTCTTCAACGTTACATTACAGAGTCTTCCGTTTGATCTCCTCAACTTCTTGGAGCTTCAATCCGATCGGTCATGGCGGAGATTGAGCTGAAAACAGCACCTGCTGACTTCCGATTCCCTACTACAAATCAAACCAGGCATTGTTTCACTCGTTACATTGAGTTCCACAGGTGCCTGACAGCAAAGGGTGAGGAGTCTGGTGAATGTGAGAGATTTGCAAAGTACTATCGTGCTCTTTGCCCTGGTGAATGGGTCGAAAATGGAATGAGCAGAGGGAGAACGGCACTTTCCCCGGCCCTCTTTGAAAGATGTTCATCCCCCGGAAAGCACAGATTGTTAATTGACGTTGTTCTTGTATTAACATACAGTTCGAACCATTGAATTTCTTATGTCCTAAAATGACGATACATACACTCGTTATATGGATTATAATTTTGAAGGTTTAAGAAAA

WMU42945 alignment (191-211)

WMU42945 5'GUGCCU-GACAGCAAAGGGUG 3'

||||x:x||||||x||||||:

cuc-miR4 3'CACGAGUCUGUCGGUUCCCAU 5'

**b) Target in pepo**

**PU084326 sequence (cytochrome c oxidase)**

ACGTCGGGGTAGTTCTATTGGTCCGTAGTATTTGTAGTCTTGTAAAACAGCACCTGCTGACTTCCGATTCCCTACTACAAATCAAACCAGGCACTGTTTCACTCGTTACATTGAGTTCCACAGGTGCCTAGACAGCAAAGGGTGAGGAATCTGGTGAATGTGAGAGA

PU084326 alignment(124-144)

PU084326 5'GUGCCUAGACAGCAAAGGGUG 3'

||||x:|||||||x||||||:

cuc-miR4 3'CACGAGUCUGUCGGUUCCCAU 5'
