# Supplementary material for: Characterization of the small RNA component of leaves and fruits from four different cucurbit species
Source: BMC Genomics. 2012 Jul 23;13:329. doi: 10.1186/1471-2164-13-329 (PMC3431224; doi:10.1186/1471-2164-13-329)
Supplement: Additional file 3 — Table 2. Primers used for real-time RT PCR analysis. [file 1471-2164-13-329-S3.doc]

Supp Table 2: primers used for real-time RT PCR analysis

| cuc-miR156-RT | GTCGTATCCAGTGCAGGGTCCGAGGTATTCGCACTGGATACGACGTGCTC |
| --- | --- |
| cuc-miR159-RT | GTCGTATCCAGTGCAGGGTCCGAGGTATTCGCACTGGATACGACTAGAGC |
| cuc-miR164-RT | GTCGTATCCAGTGCAGGGTCCGAGGTATTCGCACTGGATACGACAGCACG |
| cuc-miR166-RT | GTCGTATCCAGTGCAGGGTCCGAGGTATTCGCACTGGATACGACGGGGAA |
| cuc-miR171-RT | GTCGTATCCAGTGCAGGGTCCGAGGTATTCGCACTGGATACGACGATATT |
| cuc-miR156-FP | GCGGCGGTGACAGAAGAGAGT |
| cuc-miR159-FP | CGGCGGTTTGGATTGAAGGGA |
| cuc-miR164-FP | CGGTGGAGAAGCAGGGCA |
| cuc-miR166-FP | TCGCTTCGGACCAGGCTTCA |
| cuc-miR171-FP | TTCCTTGATTGAGCCGCGCC |
| cuc-miR4-RT | GTC GTA TCC AGT GCA GGG TCC GAG GTA TTC GCA CTG GAT ACG ACG TGC TC |
| s3509497-RT | GTC GTA TCC AGT GCA GGG TCC GAG GTA TTC GCA CTG GAT ACG ACA GCC GA |
| s908659-RT | GTC GTA TCC AGT GCA GGG TCC GAG GTA TTC GCA CTG GAT ACG ACC TGC CA |
| cuc-miR4-FP | GGC GGT ACC CTT GGC TGT CT |
| s3509497-FP | GCG GCG GCC CAG TCC CGA ACC CG |
| s908659-FP | GCG GCG GAC AGG GTA TTG TAA G |
